# Neurocognitive dysfunction in adolescents with recent onset major depressive disorder: a cross-sectional comparative study

**DOI:** 10.1007/s00787-024-02599-0

**Published:** 2024-11-06

**Authors:** Olga Bienek, Kelly Allott, Linda Antonucci, Alessandro Bertolino, Carolina Bonivento, Stephan Borgwardt, Paolo Brambilla, Katharine Chisholm, Udo Dannlowski, Theresa K. Lichtenstein, Joseph Kambeitz, Lana Kambeitz-Ilankovic, Nikolaos Koutsouleris, Rebekka Lencer, Siân Lowri Griffiths, Eleonora Maggioni, Eva Meisenzahl, Christos Pantelis, Marlene Rosen, Stephan Ruhrmann, Raimo K. R. Salokangas, Alexandra Stainton, Marian Surmann, Rachel Upthegrove, Julian Wenzel, Stephen J. Wood, Georg Romer, Jörg Michael Müller, the PRONIA Consortium, Olga Bienek, Carolina Bonivento, Udo Dannlowski, Alexandra Stainton, Marian Surmann, Shalaila Haas, Alkomiet Hasan, Claudius Hoff, Ifrah Khanyaree, Aylin Melo, Susanna Muckenhuber-Sternbauer, Yanis Köhler, Ömer Öztürk, Nora Penzel, David Popovic, Adrian Rangnick, Sebastian Saldern, Rachele Sanfelici, Moritz Spangemacher, Ana Tupac, Maria Fernanda Urquijo-Castro, Johanna Weiske, Antonia Wosgien, Camilla Krämer, Karsten Blume, Dennis Hedderich, Dominika Julkowski, Nathalie Kaiser, Thorsten Lichtenstein, Ruth Milz, Alexandra Nikolaides, Tanja Pilgram, Mauro Seves, Martina Wassen, Christina Andreou, Laura Egloff, Fabienne Harrisberger, Ulrike Heitz, Claudia Lenz, Letizia Leanza, Amatya Mackintosh, Renata Smieskova, Erich Studerus, Anna Walter, Sonja Widmayer, Chris Day, Sian Lowri Griffiths, Mariam Iqbal, Mirabel Pelton, Pavan Mallikarjun, Ashleigh Lin, Alexander Denissoff, Anu Ellilä, Tiina From, Markus Heinimaa, Tuula Ilonen, Päivi Jalo, Heikki Laurikainen, Antti Luutonen, Akseli Mäkela, Janina Paju, Henri Pesonen, Reetta-Liina Säilä, Anna Toivonen, Otto Turtonen, Sonja Botterweck, Norman Kluthausen, Gerald Antoch, Julian Caspers, Hans-Jörg Wittsack, Giuseppe Blasi, Giulio Pergola, Grazia Caforio, Leonardo Fazio, Tiziana Quarto, Barbara Gelao, Raffaella Romano, Ileana Andriola, Andrea Falsetti, Marina Barone, Roberta Passiatore, Marina Sangiuliano, Ana Beatriz Solana, Manuela Abraham, Timo Schirmer, Carlo Altamura, Marika Belleri, Francesca Bottinelli, Adele Ferro, Marta Re, Emiliano Monzani, Maurizio Sberna, Giampaolo Perna, Maria Nobile, Alessandra Alciati, Armando D’Agostino, Lorenzo Del Fabro, Matteo Balestrieri, Giuseppe Cabras, Franco Fabbro, Marco Garzitto, Sara Piccin

**Affiliations:** 1 Clinic for Child and Adolescent Psychiatry, Psychotherapy and Psychosomatic Medicine, University Hospital Muenster, Schmeddingstrasse 50, 48149 Muenster, Germany; 2 Institute for Translational Psychiatry, University Hospital Muenster, Münster, Germany; 3 Department of Psychiatry and Psychotherapy, Faculty of Medicine and University Hospital, University of Cologne, Cologne, Germany; 4 Clinic for Psychiatry and Psychotherapy, University Hospital Luebeck, Lübeck, Germany; 5 Pasian Di Prato (UD), Scientific Institute IRCCS ‘Eugenio Medea’, Polo FVG, 33037 Udine, Italy; 6 Department of Psychiatry and Psychotherapy, Ludwig-Maximilians-University, Munich, Germany; 7 Centre for Youth Mental Health, University of Melbourne, Parkville, Australia; 8 Institute for Mental Health, University of Birmingham, Birmingham, UK; 9 School of Psychology, Sussex University, Brighton, UK; 10 Department of Education, Psychology, and Communication, University of Bari Aldo Moro, Bari, Italy; 11 Department of Electronics, Information and Bioengineering, Politecnico Di Milano, Milan, Italy; 12 Department of Psychiatry, Psychiatric University Hospital, University of Basel, Basel, Switzerland; 13 Department of Pathophysiology and Transplantation, University of Milan, Milan, Italy; 14 Department of Neurosciences and Mental Health, Fondazione IRCCS Ca’ Granda Ospedale Maggiore Policlinico, Milan, Italy; 15 Department of Psychiatry and Psychotherapy, Medical Faculty, Heinrich-Heine University, Düsseldorf, Germany; 16 Department of Psychiatry, University of Turku, Turku, Finland; 17 Melbourne Neuropsychiatry Centre, University of Melbourne and Melbourne Health, Melbourne, Australia; 18 Department of Basic Medical Science, Neuroscience and Sense Organs, University of Bari Aldo Moro, Bari, Italy; 19 Max-Planck Institute of Psychiatry, Munich, Germany; 20 Institute of Psychiatry, Psychology and Neuroscience, King’s College London, London, UK; 21 Orygen, Melbourne, Australia; 22 School of Psychology, University of Birmingham, Birmingham, UK; 23 University of Oxford, Oxford, UK

**Keywords:** Neurocognitive dysfunction, PRONIA, Recent onset, Major depressive disorder, Adolescent

## Abstract

The aim of this study was to examine the neurocognitive deficits associated with the first episode of major depressive disorder (recent onset depression, ROD) in adolescents as compared to adult patients. Cross-sectional neurocognitive data from the baseline assessments of the PRONIA study with *N* = 650 (55.31% females) were analyzed. Based on a principal component analysis of eleven neurocognitive tests, we constructed an overall neurocognitive performance (NP) score. We examined mean score differences in NP between the groups of healthy controls (HC) and ROD and between adolescents (15–21 years) and adults (22–40 years) within a GLM approach. This accounts for unbalanced data with focus on interaction effects while controlling for effects of medication and educational years. Our results show lower NP for the ROD as compared to the HC group (d = − 0.29, *p* = .046) and lower NP for the adolescent group as compared to the adult group (d = − 0.29; p < .039). There was no interaction between these two group effects (F = 1.11; p = .29). Our findings suggest that the detrimental effect of ROD on neurocognitive functioning is comparable in adolescent and adult patients, since lower scores in adolescent patients are explained by effects of age and education. Neurocognitive impairment is an under addressed issue in clinical treatment guidelines for adolescent MDD. We suggest efficient monitoring in clinical practice by using an aggregate of the Digit Symbol Substitution Test and the Trail Making Test B, which highly correlated with the overall score of NP (r = 0.82).

**Supplementary Information:**

The online version contains supplementary material available at 10.1007/s00787-024-02599-0.

## Introduction

Major depressive disorder (MDD) represents one of the most common psychiatric diseases and has debilitating effects on communities worldwide. In Europe, it has a point prevalence of 6.38% [1]. While the first onset of MDD often occurs between 20–30 years of age, it also peaks in adolescence, during which the 1 year prevalence is estimated at 8% [2, 3].

In addition to the mood-altering symptoms of MDD, neurocognitive impairments are very common and have been identified as core symptoms of MDD [4–6]. Commonly MDD affects multiple cognitive domains, including working memory, attention, and psychomotor processing speed, occurring in up to 30% of patients [7–9]. Patients report subjective symptoms such as problems with concentration and memory, often causing a loss of self-esteem in the context of working performance, loss of productivity at work and loss of employment [10, 11]. Neurocognitive deficits are associated with poor treatment response and poorer social and occupational outcomes [11–14]. Detecting neurocognitive impairment is therefore highly relevant for treatment, as neurocognitive impairment has been shown to persist in MDD for several years [10].

### Adolescence and neurocognitive impairment

Despite this well-documented relevance of neurocognitive impairment for the overall sequelae of MDD, little attention has been paid to this aspect of MDD regarding diagnostic and treatment guidelines of MDD in the field of child and adolescent psychiatry.

There are few studies that have examined neurocognitive impairment in young adults and adolescents with MDD [6, 15]. They have shown that an early onset of depression in adolescence is associated with a worse prognosis, more severe symptoms and is more resistant to treatment than adult onset MDD [16–19]. It also increases the risk for relapse, and each episode increases the risk of further recurrence [20–23].

Impairments in executive functions in adolescent MDD seem to represent a state which correlates with the severity of the depressive episode and fluctuates accordingly [24, 25]. These results are consistent with findings in adult patients with MDD. Some findings suggest that there are differences in the persistence of neurocognitive impairments between adults and adolescents with MDD. According to a study by Maalouf and colleagues [24], adolescents with neurocognitive impairment during an MDD episode were unimpaired after remission from their affective symptoms, while in the adult patient group the neurocognitive impairments persisted. However, samples of adult patients with MDD in previous studies were confounded with longer durations of disease. Therefore, it is impossible to determine wherever such differences are due to the shorter duration of disease in previous adolescent samples or the greater plasticity of the juvenile brain.

### Neurocognitive impairment as treatment target

Regarding treatment recommendations addressing neurocognitive impairment in MDD, the evidence is sparse. Cognitive remediation is a common therapy element in diseases such as e.g. schizophrenia, but it is not a standard recommendation in the treatment of MDD. Some studies suggest that it might have beneficial effects in the treatment of MDD [26, 27]. Neurocognitive impairments may interfere with the efficacy of other therapies, e.g. cognitive behavioral therapy, which requires a certain level of cognitive functioning [28].

Regarding the use of medication, neurocognitive performance (NP) is usually not a primary outcome target in therapy studies, especially those that include adolescents with MDD. SSRIs (selective serotonin reuptake inhibitors) and SNRIs (serotonin and norepinephrine reuptake inhibitors) have been shown to correlate with improvements in working memory and psychomotor speed, and executive function such as inhibition of automated responses and planning [29, 30].

### Objectives of the study

Our main research question was whether differences in neurocognitive impairments exist between adolescent (15–21 years old) and adult (22–40 years old) patients presenting with a first episode of MDD. Studies have shown that adolescent brain maturation continues up to an age of 24 years [31]. However, in clinical settings, patients above 18 years of age are mostly treated as adults. We applied the definition used by both the American Academy of Pediatrics and the German medical system, which allows for medical treatment in pediatric health care up to the age of 21 years [32]. Therefore, our age groups were formed based on different neurodevelopmental stages and on a differing access to medical and mental health care. We hypothesized that an onset of MDD in the critical neurodevelopmental stage of adolescence may have more detrimental effects on neurocognitive function than a later onset of MDD, due to a longer period of unaffected brain development into adulthood. We also examined the various domains of neurocognitive function in which neurocognitive impairments may occur. Our sample was well suited to this objective, as the PRONIA data exclusively included individuals with first onset MDD. This notably allowed us to rule out cumulative effects that are inherent in a longer duration of the disease in adult patients.

## Methods

### Sample

For our study, we used the data derived from the PRONIA project (Personalized Prognostic Tools for Early Psychosis Management), a study designed to profile and predict the outcome of patients with early detected risks for and first episodes of psychosis and MDD. We combined the discovery (N = 441) and replication subsamples (N = 311) of the PRONIA sample into one data set (N = 752) in the age range of 15–40 years old [33].

For analysis, we only included the cross-sectional data of participants in the PRONIA sample classified as healthy controls (HC) or recent onset depression (ROD) at baseline. For HC, exclusion criteria included a diagnosed axis 1 psychiatric disorder, having a first degree relative with an affective or non-affective psychotic disease and taking any antipsychotic or psychotropic medications (any time in the month preceding the trial or more than 5 times a year). Participants in the ROD group had to fulfill the criteria for MDD for the first time within the last 3 months, as described by the Structured Clinical Interview for DSM-IV-TR (SCID). Exclusion criteria included prior MDD episodes predating the current episode and a duration of the episode of more than 24 months, as well as an IQ lower than 70 points. Participants taking antipsychotic medication above a certain dosage were excluded by the original study. Additionally, we excluded participants taking stimulating medication (e.g. methylphenidate). We retained the participants taking antidepressants or any centrally sedating medication, i.e. predominantly neuroleptics or benzodiazepines (see Appendix A for a list of substances) [33]. This distribution of patients with medication is described in Table 1. Because medication may influence NP, it is treated as a covariate within our analysis. Furthermore, we excluded N = 9 participants within the HC group with increased depression scores in the BDI-II above the cut-off suggested by Dolle et al. (2012) from our analysis to strengthen the internal validity of the planned comparison [34]. N = 89 participants were excluded due to missing data. Our analysis is based on N = 650 subjects which show singular missing data on different variables.

**Table 1:** Clinical and demographic sample description

	Total	HC		ROD	
		Adolescent	Adult	Adolescent	Adult
N	650	56	356	22	216
Female, N (%)	55.31	53.57	60.11	54.55	50.93
Mean years of age (SD)	28.40 (6.23)	19.82 (0.97)	29.69 (5.55)	19.59 (1.62)	29.38 (5.90)
BDI-II mean score (SD)	11.61 (14.24)	6.07 (4.75)	2.89 (3.65)	24.29 (17.39)	26.57 (13.60)
Mean years of education (SD)	15.42 (3.16)	11.27 (1.46)	16.62 (2.72)	10.94 (1.76)	14.65 (2.91)
Medication (% by column)					
no medication %	69.38	100	96.35	68.19	17.13
Antidepressants %	27.54	0	1.40	27.27	78.78
sedating %	16.00	0	2.25	22.73	42.13
Antidepressants + sedating %	12.92	0	0	18.18	37.04

*HC* healthy control, *ROD* recent onset depression, *SD* standard deviation

### Neurocognitive measures

The eleven neurocognitive tests of the PRONIA battery, which were applied no later than 3 months after the first onset of the depressive episode, were comprised of the following subset: Digit Symbol Substitution Test, Trail Making Test A and B, the Digit Span Test, the Self-Ordered Pointing Task, the Continuous Performance Test, the Rey-Auditory Verbal Learning Test, The Verbal Fluency Tasks, The Rey-Osterrieth Complex Figure Test, The Diagnostic Analysis of Non-Verbal Accuracy. A short description for each test is given in Appendix A.

### Data preprocessing

After excluding the salience-attribution test due to missing data, we checked the remaining missing data by running our analysis without any imputation, with mean score imputation and with multiple imputation. We did not observe any differences with respect to our main hypothesis testing results. In Table 2 the descriptive test scores are reported without imputation, but the multivariate analyses are based on a mean score imputation. All neurocognitive tests scores were checked for severe deviation from a normal distribution and transformed accordingly. Scores for the neurocognitive domains and the overall score are based on neurocognitive tests in z-scored metric. To facilitate interpretation, some scores were mirrored; thus, higher scores always reflect better neurocognitive performance.

**Table 2:** Basic descriptive of eleven neurocognitive test scores for total and subsamples

	Total			Healthy control				Recent onset depression			
				Adolescent		Adult		Adolescent		Adult	
	N	M	SD	M	SD	M	SD	M	SD	M	SD
1. Digit symbol substitution test [correct responses]	648	63.82	11.49	62.00	10.15	65.68	10.81	61.24	13.14	61.47	12.24
2. Trail making test A [seconds]	650	28.90	10.57	32.62	9.42	27.31	8.81	30.94	12.74	30.35	12.66
3. Trail making test B [seconds]	649	60.14	22.28	66.10	18.72	56.20	18.91	72.52	28.56	63.85	25.96
4. Digit span test [correct responses]	647	17.31	3.97	16.45	3.30	17.98	3.80	15.41	4.00	16.63	4.19
5. Self-ordered pointing test [errors]	648	7.43	5.03	7.96	5.69	6.85	4.68	9.14	4.57	8.06	5.34
6. Continuous performance test [correct responses]	649	272.46	15.43	266.82	14.26	274.68	12.97	262.23	16.77	271.31	18.23
7. Rey-auditory _total [correct repetition]	575	59.82	7.95	56.43	8.29	61.45	7.13	56.38	8.86	58.71	8.37
8. Rey-auditory _learning [correct repetition]	576	5.14	2.34	5.79	1.94	4.92	2.38	5.48	2.82	5.24	2.30
9. Verbal fluency test [word count]	649	15.23	5.00	13.73	4.05	16.10	5.15	12.14	3.81	14.50	4.76
10. Rey-osterrieth figure [correct features]	640	34.59	2.28	35.07	1.74	34.77	2.03	34.41	2.75	34.19	2.66
11. Non-verbal accuracy [correct responses]	650	19.45	2.19	19.54	2.10	19.52	2.22	18.82	2.67	19.38	2.11

*N* number, *M* mean, *SD* standard deviation

### Data analysis strategy

We aimed to test mean score differences in NP between adolescent vs. adult and health status HC vs. ROD and their interaction (HC_adol, HC_adult, ROD_adol, ROD_adult). Generalized Linear Models with SAS GLM (SAS 9.4; Type III) were applied with a Tukey–Kramer adjustment for multiple testing and to account for unbalanced data. The number of educational years and antidepressive or sedating medication were covariate variables. We report mean scores for the tests according to the factors named above both for the original (not imputed and not adjusted for covariates) as well as the GLM estimated mean scores. These were adjusted for all remaining variables in the model and Cohens effect size D, which was based on mean score differences and pooled variance estimates. The hypothesis testing was applied on the adjusted mean score.

### Level of outcome and scoring

For a parsimony hypothesis testing we built an overall NP score based on the eleven neurocognitive tests and performed a principal component analysis [35]. The eigenvalues (3,60; 1,12) based on the eleven neurocognitive tests exceeded random eigenvalues from a parallel analysis (1,21; 1,16) only for the first eigenvalue [35]. Therefore, only one factor was retained, which represents the overall neurocognitive performance score [35]. Additionally we described the NP on domain scores according to the Cattell-Horn-Carroll (CHC) model [36] and allocated the tests to the following domains: processing speed by the Digit Symbol Substitution Test and the Trail Making Test A and B, working memory by the Digit Span Test, the Self-Ordered Pointing and the Continuous Performance Test, long-term memory by the Rey-Auditory Verbal Learning Test, word fluency by the Verbal Fluency Test. However, the Rey-Osterrieth Complex Figure Test and the Diagnostic Analysis of Non-Verbal Accuracy Test represent domains outside the CHC model, so they were assigned their own domains: visuospatial ability was defined by the Rey-Osterrieth Complex Figure Test and non-verbal social information processing, which included the correct interpretation of facial expressions, was defined by the diagnostic analysis of non-verbal accuracy. We handled their contents as separate domains and checked in an exploratory manner if specific domains were especially affected by ROD. Finally, we reported the outcome for each neurocognitive test, which was performed within 3 months of the onset of symptoms.

## Results

Basic descriptives of the eleven neurocognitive test scores for total and both main factors related to age and health, which define the subsamples ROD-Ado, ROD-Adu and HC-Ado, HC-Adu, are presented in Table 2.

### Main hypothesis testing

**Fig. 1:**
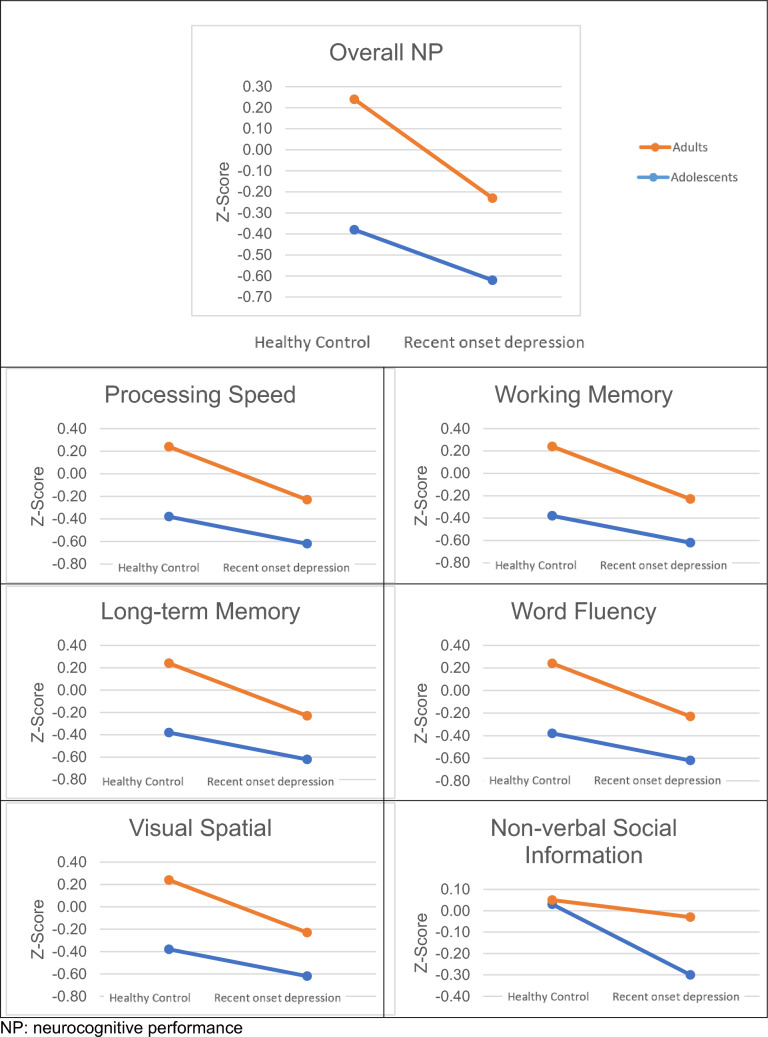
Z-standardized marginal means based on GLM estimates for the main effects of age and health status for overall NP and neurocognitive domains (CHC model) [37]. *NP* neurocognitive performance

The main hypothesis testing was conducted within a GLM approach with two classifying variables (age, health status) and the two covariates (educational years; sedating and antidepressive medication). We applied it first for the overall NP score and subsequently for each neurocognitive domain. There were statistically significant differences in neurocognitive impairment explained by the model (*F* (6, 643) = 13.00, p < 0.0001; R^2^ = 0.11). Because of unbalanced data, when using GLM Type III estimates it is recommended that any presented effect should be adjusted for all the remaining variables in the model. There, they show only incremental effects. The detailed results for the overall neurocognitive performance and domain-specific deficits are presented in Table 3. To illustrate the results visually, we present the mean z-score estimates in Fig. 1 [37]. These are adjusted for all covariates and show mean score differences in a Cohens d score metric.

**Table 3:** Results of a GLM Type III to explain overall NP for influences of age (adolescent vs. adult), health status (ROD vs. HC) and interaction including medication and educational years

	Overall		Age		Health-ROD		Age x health-ROD		Medication anti-depressants		Medication sedation		Educational years	
	F	p	F	p	F	p	F	p	F	p	F	p	F	p
Overall NP	13.00	< 0.0001	4.28	0.039	4.00	0.046	1.11	0.292	2.72	0.099	4.46	0.035	18.43	< 0.001
Processing speed	7.97	< 0.0001	2.90	0.089	2.44	0.119	1.30	0.255	2.37	0.124	4.79	0.029	7.03	0.008
Working memory	9.63	< 0.0001	3.52	0.061	3.35	0.068	0.01	0.938	1.02	0.312	0.99	0.319	21.36	< 0.001
Long-term memory	5.76	< 0.0001	4.58	0.033	0.96	0.329	3.18	0.075	2.90	0.089	0.60	0.440	2.80	0.095
Word fluency	10.37	< 0.0001	2.08	0.150	2.31	0.130	0.00	0.968	0.07	0.790	0.14	0.712	30.80	< 0.001
Visual spatial	3.54	0.0019	1.52	0.219	0.49	0.483	0.18	0.674	0.11	0.741	3.88	0.049	5.34	0.021
Non-verbal social information	1.44	0.1965	0.06	0.812	2.50	0.114	0.43	0.513	3.03	0.082	2.45	0.118	1.35	0.246

F value: F (6, 643) = 13.00, p value: p < 0.0001

As shown in Table 3, we found significant effects for both age groups, when comparing ROD patients with HC, such as that NP was reduced in the ROD groups. As to our key research question concerning a hypothesized interaction between age and clinical status, we did not find significant effects. Adults in our samples performed better throughout than adolescents irrespective of their clinical status, but there were no differences in the effects of ROD on neurocognitive impairment between adults and adolescents when we controlled for a general age effect. This was also true for the two HC groups (Fig. 1). No medication effect was found in our sample, but a strong effect of the covariate “years of education”. These overall findings apply to both the overall NP score as well as any domain of the CHC model.

The second finding is that ROD participants from both age groups performed worse than the respective HC subgroup. Our data showed that in the adult ROD group more individuals were treated with medication, albeit with partly contrary influences related to sedating and antidepressive medication. Concerning the medication, we observed that participants taking antidepressants performed better with an effect size of d = 0.23 in the overall NP score, which was particularly greater for long-term memory, non-verbal social information, and processing speed. Participants taking sedating medications showed lower scores in overall NP, which especially affected the domains processing speed, visual spatial ability, and non-verbal social information processing.

The overall NP and the varying effect sizes in the CHC domains are described in Table 4. Our results show that adults performed better across the most neurocognitive tests and domains, except for visual spatial ability, which is outside the CHC model. No single domain emerged as particularly affected.

**Table 4:** GLM estimated mean z-scores (adjusted for all included variables) and Cohens D for the main factors age (adolescent vs. adult) and health status (ROD vs. HC) [37]

	Adolescent	Adults	Cohens D	HC	ROD	Cohens D
	m	m	d	m	m	d
Overall NP	− 0.34	− 0.05	0.29	− 0.05	− 0.34	− 0.29
Processing speed	− 0.30	− 0.06	0.24	− 0.06	− 0.30	− 0.24
Working memory	− 0.29	− 0.01	0.28	− 0.01	− 0.28	− 0.27
Long-term memory	− 0.29	0.02	0.31	− 0.06	− 0.21	− 0.15
Word fluency	− 0.19	0.02	0.21	0.03	− 0.20	− 0.23
Visual spatial	0.05	− 0.13	− 0.08	0.02	− 0.09	− 0.11
Non-verbal social information	− 0.07	− 0.03	0.04	0.08	− 0.17	− 0.25

*m* mean, *D* cohens D.

An exploratory factor analysis of the data from all neurocognitive tests of our battery revealed that two subtests, the Digit Symbol Substitution Test, and the Trail Making Test B, emerged as particularly valid in predicting the overall NP score. An aggregation score of these two subtests highly correlated with the overall score of all subtests (r = 0.82). Thus, a combination of these two subtests may be suitable for both economic and valid detecting and monitoring of neurocognitive impairment in MDD.

## Discussion

This study aimed to shed light on neurocognitive impairments in adolescents with recent onset MDD as compared to adults with recent onset MDD. We hypothesized that in adolescents with ROD, who are still in a particularly vulnerable stage of brain development, the impeding effects of depression on neurocognitive impairment would be stronger than in adults. Thus, we hypothesized that adults with recent onset MDD would be more resilient due to their longer duration of unaffected brain development into adulthood prior to their first depressive episode[38].

Our research confirms that cognitive impairments were significant in the clinical ROD group across both age groups, both globally and in a range of specific domains. Thus, we observed a significantly lower NP score for the ROD group, however, the effect size was small (d = − 0.29).

In negation of our hypothesis, our results suggest that an adolescent onset of MDD does not have more detrimental effects than an onset in adulthood.

We observed a strong effect of educational years: Longer education was correlated with higher cognitive performance, both in the ROD and HC groups. We surmised that higher cognitive performance might be a result of academic exercise, which is longer and more intense for adults. But higher cognitive performance may also be a precondition for longer education, as cognitive performance is known to show a considerable overlap to measures of intelligence [39]. Furthermore, a general age effect must be considered: The common peak of cognitive performance is in early adulthood [40, 41]. Therefore, the different results in adolescents, both in the HC and the ROD subgroups, can be explained independently from the impact of the disease, only by effects of age and educational training.

Another factor is the impact of medication. Our data showed that the adult ROD subgroup received far more antidepressive medication than the adolescent ROD subgroup. After adjusting for this variable, we found that the NP scores in the adult ROD subgroup were lower. Our sampling did not allow us to examine the effect of medication in further detail.

Regarding the two suggested subtests, further research is needed to determine why and to which degree these tests are sensitive towards MDD-associated neurocognitive impairment. One hypothesis would be that both tests are fairly complex and therefore screen for a variety of impairments.

### Strengths and limitations

Our study allows a unique comparison between adolescents and adults, since all participants are experiencing their first MDD episode. This precludes any confounding due to duration of disease, which has been a major limitation in previous studies.

By design, our study does not provide insight into the origin of neurocognitive impairment. Additionally, our study lacks data that describe the participants’ neurocognitive performance prior to the onset of MDD. We cannot rule out that neurocognitive impairments were already present prior to the onset of MDD or might have contributed to the development of MDD. Our design also did not allow for longitudinal analyses of the long-term development of NP of the ROD participants. The effects of medication were examined solely for the participants taking sedating or antidepressive medication. Because of the strong covariation of age and medication the analysis probably could not fully entangle confounding effects. In the future, more research is needed on the specific effects of pharmacological treatment on NP.

### Implications for future research and clinical practice

Diagnostic and treatment standards for adolescent MDD to date have mainly focused on detecting and treating affective symptoms, as well as on addressing impairments in social functioning. Our study shows that adolescents with MDD have similar impairments in their neurocognitive functions as compared to adult patients. Some studies suggest that antidepressive medication can have a positive effect on neurocognitive performance [29, 30]. Our results also point to this effect.

Our research underlines that appropriate detection and monitoring of neurocognitive impairment should be paid more systematic attention to in adolescent mental health care. More systematic research is needed so that future clinical treatment guidelines in child and adolescent psychiatry may include standardized testing and monitoring of neurocognitive functioning, as well as including specific neurocognitive training in treatment plans. This may be beneficial for educational and social achievements of adolescents with MDD and their long-term mental health prognosis, as neurocognitive impairments in MDD increase the risk of reduced long-term participation in education and employment [10, 42]. In Table 5 our explorative findings regarding the Digit Symbol Substitution Test and the Trail Making Test B suggest that these two subtests may serve as a both valid and efficient tool for detecting and monitoring neurocognitive performance. Still, further studies are needed to test and confirm their usefulness in every day clinical practice.

**Table 5:** Item loadings of eleven neurocognitive tests on the first component of a principal component analysis

Neurocognitive test	
1. Trail making test B	0.74
2. Digit symbol substitution test	0.73
3. Rey-auditory verbal learning test _learning	0.67
4. Digit span test	0.67
5. Continuous performance test	0.63
6. Trail making test A	0.58
7. Self-ordered pointing test	0.58
8. Verbal fluency test	0.47
9. Diagnostic analysis of non-verbal accuracy	0.32
10. Rey-osterrieth complex figure test	0.31
11. Rey-auditory verbal learning test_total	0.21

### Summary and conclusions

In summary, patients with ROD showed lower NP scores than HC across both age groups. Differences between the two ROD age groups were equally found between the two HC age groups. No interaction effects between clinical status and age were found. These neurocognitive impairments were visible across all neurocognitive domains we examined. No specific profiles of neurocognitive impairment in ROD groups emerged from our data. Among the tests we used in our battery, a combination of the Digit Symbol Substitution Test and the Trail Making Test B emerged as highly predictive for the overall score of neurocognitive impairment. We also found that, irrespective of their HC or ROD status, adults generally performed better in neurocognitive tasks than adolescents. This can be explained as an effect of age and cumulative educational years. Further research is needed to determine to which degree antidepressive medication can improve neurocognitive impairment in adolescent MDD patients, as our results point to a similar effect as compared to adult ROD participants. This is particularly relevant, as in current clinical practice adolescents with depressive disorders are less frequently treated with antidepressants than adult patients. More systematic attention should be paid to neurocognitive impairment in adolescent MDD both in research and clinical practice. Further research is needed to provide confirming evidence that may inform future clinical recommendations for standard tools and procedures that are suitable for detection and monitoring of neurocognitive impairment in adolescent depression.

## Supplementary Information

Below is the link to the electronic supplementary material.

Supplementary file1 (DOCX 56 KB)

## Data Availability

No datasets were generated or analysed during the current study.
