# Supplementary material for: Neurocognitive dysfunction in adolescents with recent onset major depressive disorder: a cross-sectional comparative study
Source: Eur Child Adolesc Psychiatry. 2024 Nov 6;34(6):1873–82. doi: 10.1007/s00787-024-02599-0 (PMC12198262; doi:10.1007/s00787-024-02599-0)
Supplement: Supplementary file 1 — Supplementary file1 (DOCX 56 KB) [file 787_2024_2599_MOESM1_ESM.docx]

**Appendix A**. Short description and scoring of neurocognitive tests and their location within the Cattell-Horn-Carroll (CHC) model [44].

Processing speed

1. The Digit Symbol Substitution Test (score: DSST) measures motor speed and visual perception as well as certain executive functions, such as working memory, planning and strategizing [32]. Participants are given a sheet of paper, on which they must match certain symbols to numbers according to a table presented to them [32]. The score consists of the number of properly identified symbols in a predetermined time [32]. It is included in the Wechsler Adult Intelligence Scale (WAIS-IV). There is a large variance within the speed that can be explained by age, since elderly participants are simply slower in copying the symbols, which shows that the test involves motor skills [32]. It is particularly sensitive towards processing speed, executive functioning and working memory [32].
2. Trail Making Test A (score: TMA) and
3. Trail Making Test B (score: TMB) measure speed of processing [33]. There are two parts of the test. The participant is presented with a sheet of paper and in part A must connect encircled numbers in the right order [33][34] In part B, the participant must alternately connect numbers and letters (e.g., 1, A, 2, B, 3, C) [33]. The time taken to complete the task is measured [25].

Working memory

1. The Digit Span Test (score: DST) is a subtest included in the Wechsler Adult Intelligence Scale (WAIS-IV). It measures working memory performance by having the examiner read number digits aloud and then having the participant repeat them back, adding a digit with every repetition [34]. The number of times the patient can repeat back the sequence without an error is measured. This is the forward (increasing) version; in the backward version, the subject must repeat the digits in reverse order [34].
2. The Self-Ordered Pointing Task (score: SOPT) is a task designed to evaluate executive behaviour, which regulates working memory and regulation behaviour [35]. Participants performing this task are shown a screen or sheet of paper with four different depicted objects [35]. The participant must select one of the objects, and then they are shown the next slide [35]. The participant must again pick an object, but not one they have picked previously [35]. Typically, the number of times a participant picks the same object twice is counted as errors, and the number of errors is used as a score [35].
3. The identical pairs Continuous Performance Test (score: CPT) measures selective and continuous attention and impulsive behaviour. It has been used extensively in schizophrenia research [36]. Such tests are also used in diagnostic assessment of ADHD in children as young as 8 years and older [37]. These types of tests are said to measure attention, hyperactivity, impulsivity and timing [37]. Participants are instructed to watch a screen, upon which three- or four-digit numbers appear [36]. Each time the new number is identical to the previous one, the participant must press the mouse key, which is counted as a correct response and scored [36].

Long-term memory

1. The Rey-Auditory Verbal Learning Test (score: VLT_Total) examines several different cognitive abilities simultaneously, most of which adhere to different memory structures, also including short term memory, and other executive functions [38]. A list of 15 words is read to the participant, who must then repeat it from memory [38]. This procedure is repeated several times, along with a distractor list and random words [38]. In some versions, the participant must recognize whenever the words were or were not from the original list [38]. The number of correct repetitions is counted and scored, [38].
2. We considered a subset of the Rey-Auditory Verbal Learning Test (score: VLT_Learning) as a separate score because this score specifically relates to the difference between the first and last repetition, therefore highlighting the participants’ learning ability [38].

Word fluency

1. The Verbal Fluency Tasks (score: VF) include two tasks, one measuring semantic fluency (in which the participant must name as many words as possible belonging to a certain category) and the other measuring phonemic fluency (in which the participant must name as many words as possible beginning with a specific letter) [39]. The standard version of the task allows the participant one minute to name as many words as possible, where the number of words the participant can think of is recorded [39]. These types of tests have been often used to measure verbal ability and executive functions [39].

Visuospatial ability

1. The Rey-Osterrieth Complex Figure Test (score: GOCF) examines visual memory and visuospatial constructional ability, therefore also measuring executive function and its mediation by the prefrontal lobe [40]. Participants are shown a picture of the Rey-Osterrieth Complex Figure and asked to copy it, then they must draw it twice more from memory within a span of 30 minutes, after which the results are graded according to the accuracy, location and organisation of the drawing, which cumulates into a score [40].

Non-verbal social information processing

1. The Diagnostic Analysis of Non-Verbal Accuracy (score: DANVA) is a test designed to examine one’s ability to correctly identify facial expressions and to name the corresponding emotions that the person is feeling [41] In the PRONIA study, the DANVA was restricted to the receptive functions and only used facial expressions. Generally, this test measures subjects’ ability to correctly interpret non-verbal social information [41]. The score in our study is the number of times the subject correctly identified the facial expression [42].

**Appendix B.** List of medication and their classification

Antidepressive medication:

Agomelatine

Amitriptiline

Amitryptiline

Bupropion

Citalopram

Clomipramine

Duloxetine

Escitalopram

Fluoxetine

Hypericum perforatum

Lithium

Mirtazapine

Moclobemid

Paroxetine

Serotonin

Sertraline

Tradozone

Trimipramine

Venlafaxine

Vortioxetine

Centrally Sedating medication:

Alprazolam

Aripiprazole

Benzodiazepine

Biperiden

Bromazepam

Chlorprotixene

Dextrometorphan

Diazepam

Diphenhydramine

Gabapentin

Lamotrigine

Lorazepam

Melatonine

Melperone

Olanzapine

Oxazepam

Paliperidone

Pipamperone

Pregabaline

Promethazine

Quetiapine

Risperidone

Tetrazepam

Triazolam

Valproate

Ziprasidone

Zolpidem

Zopiclone

**Appendix C.** The loadings pattern on the first component of a principal component analysis suggest configural measurement invariance (see Luong et al, 2023) for the subgroups health status (recent onset depression ROD vs. healthy controls), age (adolescent vs. adult), and for taking antidepressive and sedating medication.

|  | **Total** | **Recent Onset Depression** | **Healthy Control** | **Adolescent** | **Adults** | **Anti-depressive**  **Medication** | **Sedating**  **Medication** |
| --- | --- | --- | --- | --- | --- | --- | --- |
| 1. Trail Making Test B | .74 | .81 | .65 | .62 | .81 | .80 | .80 |
| 1. Digit Symbol Substitution Test | .73 | .77 | .68 | .71 | .77 | .82 | .77 |
| 1. Digit Span Test | .67 | .73 | .60 | .68 | .73 | .69 | .72 |
| 1. Rey-Auditory Verbal Learning Test _Learning | .67 | .69 | .65 | .62 | .69 | .72 | .72 |
| 1. Continuous Performance Test | .63 | .67 | .59 | .70 | .67 | .65 | .74 |
| 1. Trail Making Test A | .58 | .65 | .49 | .43 | .65 | .65 | .72 |
| 1. Self-Ordered Pointing Test | .58 | .58 | .57 | .65 | .58 | .61 | .69 |
| 1. Verbal Fluency Test | .47 | .50 | .42 | .47 | .50 | .56 | .55 |
| 1. Rey-Osterrieth Complex Figure Test | .31 | .32 | .25 | .50 | .32 | .38 | .29 |
| 1. Diagnostic Analysis of Non-Verbal Accuracy | .32 | .29 | .36 | .51 | .29 | .28 | .29 |
| 1. Rey-Auditory Verbal Learning Test _Total | .21 | .02 | .37 | .17 | .02 | .05 | .12 |

Luong, R., & Flake, J. K. (2023). Measurement invariance testing using confirmatory factor analysis and alignment optimization: A tutorial for transparent analysis planning and reporting. Psychological Methods, 28(4), 905–924. https://doi.org/10.1037/met0000441.supp (Supplemental)
